# Microbiomes of *Muricea californica* and *M. fruticosa*: Comparative Analyses of Two Co-occurring Eastern Pacific Octocorals

**DOI:** 10.3389/fmicb.2016.00917

**Published:** 2016-06-21

**Authors:** Johanna B. Holm, Karla B. Heidelberg

**Affiliations:** Division of Marine Environmental Biology, Department of Biological Science, University of Southern California, Los AngelesCA, USA

**Keywords:** microbiome, octocoral, zoanthid, amplicon sequencing, microbial diversity, MiSeq

## Abstract

Octocorals are sources of novel but understudied microbial diversity. Conversely, scleractinian or reef-building coral microbiomes have been heavily examined in light of the threats of climate change. *Muricea californica* and *Muricea fruticosa* are two co-occurring species of gorgonian octocoral abundantly found in the kelp forests of southern California, and thus provide an excellent basis to determine if octocoral microbiomes are host specific. Using Illumina MiSeq amplicon sequencing and replicate samples, we evaluated the microbiomes collected from multiple colonies of both species of *Muricea* to measure both inter- and intra-colony microbiome variabilities. In addition, microbiomes from overlying sea water and nearby zoanthids (another benthic invertebrate) were also included in the analysis to evaluate whether bacterial taxa specifically associate with octocorals. This is also the first report of microbiomes from these species of *Muricea.* We show that microbiomes isolated from each sample type are distinct, and specifically, that octocoral species type had the greatest effect on predicting the composition of the *Muricea* microbiome. Bacterial taxa contributing to compositional differences include distinct strains of *Mycoplasma* associated with either *M. californica* or *M. fruticosa*, an abundance of *Spirochaetes* observed on *M. californica*, and a greater diversity of γ-Proteobacteria associated with *M. fruticosa*. Many of the bacterial taxa contributing to these differences are known for their presence in photosymbiont-containing invertebrate microbiomes.

## Introduction

Corals and their microbiomes (including microbial eukaryotes, bacteria, archaea, and viruses) together comprise the entire metaorganism (Bosch and McFall-Ngai, 2011), and these symbiotic associations are critical to host survival (Rosenberg et al., 2007; Bourne and Webster, 2013). Members of these microbiomes contribute to shared metabolic functions, such as nutrient acquisition, environmental sensing, and protection from disease (Rohwer et al., 2002; Reshef et al., 2006; Vega Thurber et al., 2009; Thomas et al., 2010). Despite the perceived importance, clear factors shaping a coral’s microbial composition have yet to be fully discerned (Bourne and Webster, 2013).

Most coral microbiome studies have focused on reef-building scleractinian corals. Few studies have examined the diversity and composition of gorgonian-associated microbial communities (Brück et al., 2007; Webster and Bourne, 2007; Gray et al., 2011; Bourne et al., 2013; Correa et al., 2013) and fewer have examined those of temperate gorgonians (La Rivière et al., 2013; Vezzulli et al., 2013). The gorgonian microbiomes described to date were targeted using culture-based, fingerprinting, and/or clone library analyses [except Bourne et al., 2013, which uses 454 pyrosequencing]. Such strategies are capable of providing taxonomic resolution but capture only a small portion of total microbial diversity. Additionally, there are no studies to our knowledge that statistically compare gorgonian-associated prokaryotic diversity to the surrounding sea water, other benthic organisms, or co-occurring sister species, which would highlight unique niches provided by each host type from the same environment.

The previous studies described above show gorgonian-associated microbial communities from various environments, including the Great Barrier Reef, the deep-sea, and the Mediterranean Sea, were dominated by *Gammaproteobacteria* (i.e., *Endozoicomonas*) or *Tenericutes* (especially *Mycoplasma*). However, it is intriguing that similar bacterial classes dominate the observed microbiomes of these gorgonian genera located in vastly different (and, in some cases, extreme) marine environments, and raises the question of how much the environment influences microbiome composition. This highlights a need to more deeply examine the gorgonian-associated prokaryotic community with high sampling effort and biological replication, in order to more fully characterize prokaryotic diversity.

We evaluated the microbiomes of two species of *Muricea*, a genus of azooxanthellate (Van Oppen et al., 2005) gorgonian octocorals found throughout the tropical and temperate eastern Pacific and western Atlantic oceans. *Muricea californica* and *M. fruticosa* co-exist in the temperate kelp forests of southern California and are easily distinguished from each other by the colors of their polyps, golden-orange or white, respectively (Grigg, 1970). Their overlapping habitats, similar abundances, colony structures, and general life histories make these species of *Muricea* suitable for comparison. Multiple colonies of both species were sampled with biological replication to determine the mean microbiome compositions for each colony. To test the specificity of microbial associations, we attempted to maximize intra-colony and inter-colony microbiome variations by purposely sampling colonies from different depths. To further evaluate the presence of gorgonian-specific associations, we also examined the microbiomes of nearby *Parazoanthus lucificum* (zoanthid, suborder Macrocnemia) colonies in addition to the surrounding sea water. Azooxanthellate *P. lucificum* (also referred to as *Savalia lucifica*, Sinniger et al., 2013), named for the brilliant bioluminescence it emits, was specifically chosen as a comparative organism because it occupies similar space as *Muricea* colonies in both the water column and the benthos due to its life-history trait of infecting and overgrowing *M. californica* colonies (Cutress and Pequegnat, 1960). To our knowledge, only one other zoanthid (suborder: Brachycnemina) microbiome has been described (Sun et al., 2014). Herein, we characterize and compare deeply sequenced microbiomes of *M. californica, M. fruticosa*, and *P. lucificum*, and in addition, examine gorgonian-associated microbes via light and fluorescence microscopy from mucus and polyp tissue to better understand bacterial micro-niches.

## Materials and Methods

### Sample Collection

All sample collections were made in accordance with CA-DFW Scientific Collecting Permit #12734, issued to J. Holm. *M. californica* (Mc) and *M. fruticosa* (Mf) samples were collected in replicate in addition to nearby samples of the zoanthid, *P. lucificum* (Pl). Samples were collected from a rocky wall of Santa Catalina Island, CA (33°26′ 53.9″ N, 118°28′ 42.3″ W) midday on October 14, 2013. *Muricea* species were distinguished using morphological characters previously described (Grigg, 1970).

For each Mc and Mf species, subsamples from three colonies from different depths were sampled *in situ* (range: 8–16 m depth). Collection depth is indicated in the sample’s name, following the replicate branch number. *Muricea* colonies are <1 m wide so, the distances between branch replicates from the same colony were no more than 1 m (i.e., colonies were no more than 1 m wide). Colonies from similar depths were >1 m but no more than 3–4 m apart. Sampling techniques to reduce contamination were employed; samples were captured in 50 mL conical tubes without handling. Additionally, single branches from two Pl colonies from depths 9 and 16 m growing adjacent to the 11–12 m gorgonian colonies, were collected. The zoanthid samples were cut and collected in a similar manner as *Muricea* samples. One liter of SW was collected from 12 m depth ca. 2 m away from the rocky wall. Upon returning to the boat, samples were immediately processed as follows: ethanol-wiped forceps were used to remove a collected sample from the conical tube and the sample was dipped multiple times in 0.02 μm-filtered sea water to remove unattached debris and contaminating overlying sea water. Samples were immediately placed in Ambion RNAlater as per manufacturer’s instructions (Thermo Fisher Scientific, Waltham, MA, USA) and stored at 4°C for 3 weeks until DNA extraction.

### Total DNA Extraction and PCR Amplification of 16S rRNA

Prior to extraction, samples were processed to remove RNAlater as per the manufacturer instructions. Briefly, each sample was aseptically removed from RNAlater, weighed, and a 50 mg subsample was placed in 450–500 mL 1× PBS, pH 8.0. Samples were centrifuged for 1 min at 4000 × *g*, and the supernatant containing residual RNAlater was carefully removed. Pellets were subsequently processed using the PowerPlant Pro DNA Isolation kit (MO BIO Laboratories, CA, USA) according to the manufacturer instructions using a TissueLyser II (Qiagen, Valencia, CA, USA) at 30 Hz for 10 min. Due to large amounts of mucous, the Sj sample and 50 mg of each Pl sample were first ground using liquid nitrogen and a sterilized mortar and pestle prior to DNA isolation.

Bacterial and Archaeal V4–V6 regions of the 16S rRNA gene were amplified using primers A519F (CAGCMGCCGCGGTAA; Wang and Qian, 2009) and 1061R (CRRCACGAGCTGACGAC; Andersson et al., 2008) from probeBase (Loy et al., 2007). Final amplification reaction volumes were 25 μL and contained 1× Q5 High-Fidelity 2× Master Mix (New England Biolabs, Ipswich, MA, USA), 100 ng template, and 1 μM of each primer. Reactions were run with a single denaturation step at 98°C for 30 s followed by 30 cycles at 98°C for 30 s, 59°C for 15 s, and 72°C for 30 s and completed with a final extension step of 72°C for 2 min. DNA from a previously collected, typical water sample was also amplified using this protocol and running the PCR for 35 cycles. Amplified DNA was visualized on a 1% agarose gel using SYBR Gold Nucleic Acid Stain, purified using DNA Clean & Concentrator-5 kits (Zymo Research, Irvine, CA, USA), and eluted with 6 μL nuclease-free water (Thermo Fisher Scientific, Waltham, MA, USA). Amplicons were first quantified on a Nanodrop (Thermo Fisher Scientific, Waltham, MA, USA) to ensure the 260/280 absorbance ratio was near 1.8 and then quantified again using the Quant-iT dsDNA HS Assay kit (Invitrogen, Carlsbad, CA, USA) and measured on a Qubit fluorometer (Invitrogen).

### Illumina Library Preparation and High-Throughput Sequencing

Amplicon libraries were prepared for Illumina MiSeq multiplex paired-end sequencing using NEBNext Ultra DNA Library Prep kit and the NEBNext Multiplex Oligos for Illumina Index Primers Sets 1 and 2 (New England Biolabs, Ipswich, MA, USA). Twenty nanograms of amplicon DNA were used for each library preparation reaction. Ampure Beads (Beckman Coulter, Indianapolis, IN, USA) were used for all DNA purification steps following the manufacturer instructions. Adaptor-ligated and indexed samples were visualized for purity and quantification using an Agilent 2100 Bioanalyzer (Agilent, Santa Clara, CA, USA). Twenty samples with final molar concentrations >1 nM were submitted to the UC Davis Genome Center (Davis, CA, USA) for paired-end, multiplex sequencing on the Illumina MiSeq platform using the MiSeq Reagent Kit v3 (Illumina, San Diego, CA, USA). Cluster generation, sequencing (600 cycles), image processing, demultiplexing, and quality score calculations were all performed on the MiSeq 500 platform (Illumina). Raw read data have been submitted to the NCBI Sequence Read Archive under BioSample Accession numbers SAMN03203155-SAMN03203174 within BioProject Accession number PRJNA268033. We further filtered reads for quality using the IlluminaClip (default settings), and Sliding Window (4 bases, average quality score of >25) options in Trimmomatic (Bolger et al., 2014).

Sequence assembly and quality control described here were performed using mothur (v 1.32.1-v.1.33.0; Schloss et al., 2009). The MiSeq v3 reagent kit (Illumina) produced read lengths ca. 300 bp. Paired-end reads that included the V4–V6 variable regions and overlapped in the 16S ribosomal RNA gene C45 region were processed. Sequence contigs shorter than 501 bp with >10 ambiguous bases and homopolymers >10 bases were removed. Sequences were aligned to the SILVA SSU Ref Non-redundant (NR) 119 database (Quast et al., 2013) and trimmed to equal alignment length (608 bp with gaps, 552 bp mean sequence length). Chimeric sequences were removed (39,076 sequences or 6.4%, UCHIME (Edgar et al., 2011), and the remaining sequences (606,679 unique) were taxonomically classified as described in (Wang and Qian, 2009) using the SILVA SSU Ref NR 119 formatted for mothur.

### Sequence Binning and Phylogenetic Analyses

Sequencing and assembly generated an average of 22,685 assembled 16S rRNA gene fragments per sample (range: 11,063–33,481). Sequences were binned into operational taxonomic units (OTUs) of 97% identity using the Average Neighbor method (Schloss and Westcott, 2011) and resulted in 5594 OTUs. Of these, 3736 were singletons and an additional 70 OTUs were characterized as chloroplasts and were culled from further analyses. Samples were randomly normalized to contain an equal number of sequences for comparative purposes (determined by the sample containing the fewest sequences, Mc_1_12m, 10,448 sequences). Alpha-diversity statistics including Good’s Coverage Estimator and the mean number of observed OTUs were then calculated using mothur (v. 1.33.3) and compared using Student’s *t*-test.

Remaining Bray–Curtis Dissimilarity Indices of the abundances of remaining OTUs were calculated to assess beta-diversity, and visualized by hierarchical clustering (hclust) using the average neighbor method and nMDS analyses (metaMDS), with R and the Vegan package (Oksanen et al., 2013). Statistical differences in microbial community composition between different samples (gorgonian vs. non-gorgonian), species (Mc vs. Mf), colonies, and depth (8–9 m vs. 10–12 vs.16 m) were tested for using the adonis function (PERMANOVA test) with 999 permutations, also in Vegan. The OTUs contributing the most to the observed clusters were determined using the Vegan function simper, and verified using the same function on Primer-E (Clarke, 1993). OTU consensus taxonomies were obtained using the SILVA SSU Ref NR 119 database, and OTU sequence representatives were extracted in mothur. OTUs that were unclassifiable beyond Phylum or Class level were examined more closely using the NCBI GenBank NR and 16S ribosomal RNA reference (Bacteria and Archaea) databases (October 2014) and the BLASTN algorithm (Altschul et al., 1990). Reference sequences with the highest percent identity and lowest *e*-values were used to construct a phylogenetic tree (the number of top matches, highest percent identities, and *e*-values varied across OTU representatives). Maximum-likelihood phylogenetic relationships of the OTU representative sequences were assessed using ClustalW (Thompson et al., 2002) to align sequences, and maximum-likelihood trees calculated using the Jukes–Cantor model of substitution (Jukes and Cantor, 1969) with 1000 bootstrap replicates (Geneious v.5.6.4).

Relative abundances of normalized microbiomes were compared across samples bacterial classes using a Bubble Plot in MS Excel. Pie charts of the same data were composed excluding major OTUs (determined from the SIMPER analyses) for each species of *Muricea* to examine underlying diversity. The OTU representative sequence data have been submitted to the GenBank database under accession numbers KP174126-KP174134.

### Microscopic Observations and Mucus Production

Endozoic cells were imaged *in vivo* using a BX51 epifluorescent microscope equipped with a DP70 digital camera (Olympus) using the “chlorophyll” filter set (excitation: 480 nm, emission: 660 nm).

Four colonies of Mc and two colonies of Mf were collected and maintained in tanks with unfiltered, flow through sea water, for four weeks during June 2012. Colonies were exposed to natural light conditions. All colonies were examined for the percentage of the total colony that was visibly covered with mucus. When visible, samples of mucus were collected with a sterile syringe, incubated with SYBR Green nucleic acid stain as per the manufacturer’s instructions (Bio-Rad), and examined using an Olympus BX5100 epi-fluorescent microscope.

## Results

**FIGURE 1:**
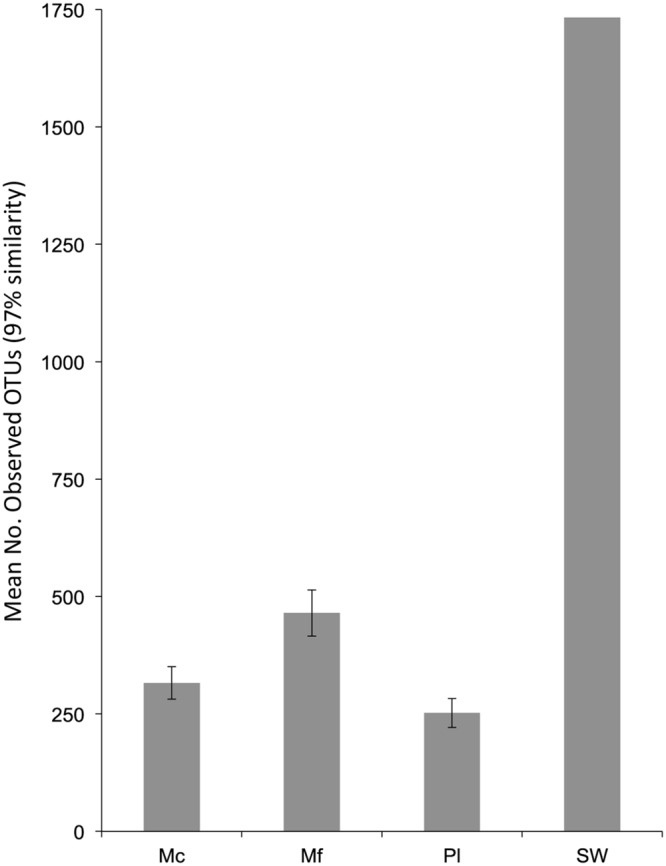
**The mean observed number of OTUs per sample from the non-normalized dataset.** *N* for samples are as follows: Mc = 7, Mf = 9, Pl = 2. Error bars are standard error of the mean.

Good’s coverage estimated a mean of 96.3% for all organismal samples (range: 92.3–98.3%), indicating adequate sampling effort (Supplemental Table S1). SW had the lowest coverage (83.4%) but the greatest number of observed OTUs, while all organism-associated microbiomes exhibited less diversity (**Figure 1**). Normalized Mf samples consistently had more observed OTUs than Mc (*t*_15_ = 2.44, *p* = 0.027).

**FIGURE 2:**
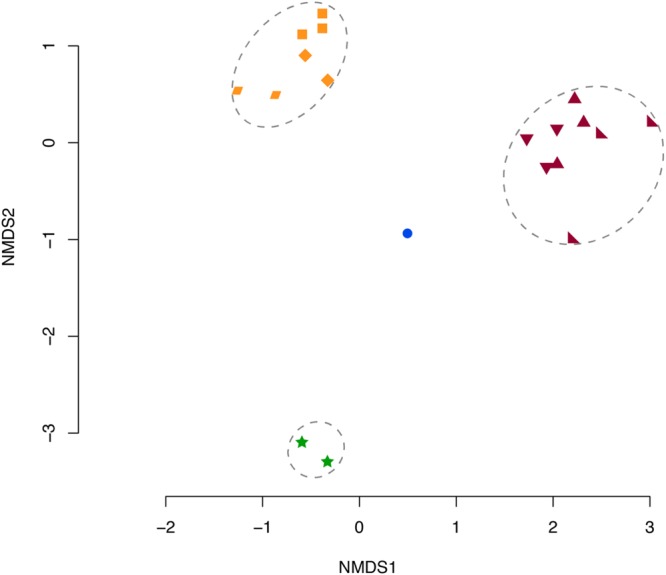
**NMDS of Bray–Curtis Dissimilarity Indices for each sample microbiome.** Orange: Mc 9 m (◆), 12 m(▰), 16 m(⬛). Purple: Mf 8 m(▲), 10 m(◣), 11 m(▼). Blue: SW (⚫), and Green: Pl (stars). Dotted circles indicate significant clusters at 20% similarity. Mc: *Muricea californica*, Mf: *Muricea fruticosa*, SW: sea water, Pl: *Parazoanthus lucificum*.

The microbiomes produced distinct sample-specific clusters whereby taxonomic assemblages from each sample were 20% similar to the other samples within the cluster (**Figure 2**). OTUs primarily contributing to the observed clusters in **Figure 2** were OTUs 1, 3, 4, and 7 according to the SIMPER analysis.

**FIGURE 3:**
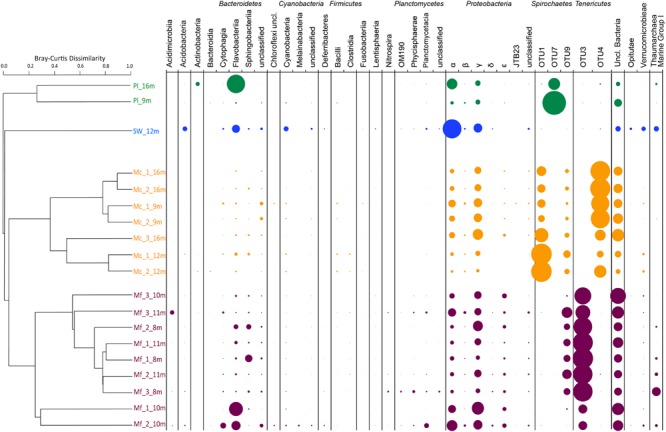
**Bray–Curtis Dissimilarity Indices as hierarchical clustering dendrogram.** Microbiomes summarized as taxa at the Class level per sample. Bubble size indicates relative abundance of taxa within a sample. Pl: *Parazoanthus lucificum*, SW: sea water, Mc: *Muricea californica*, Mf: *Muricea fruticosa*. Sample names are “species_biological replicate_depth of collection”.

**FIGURE 4:**
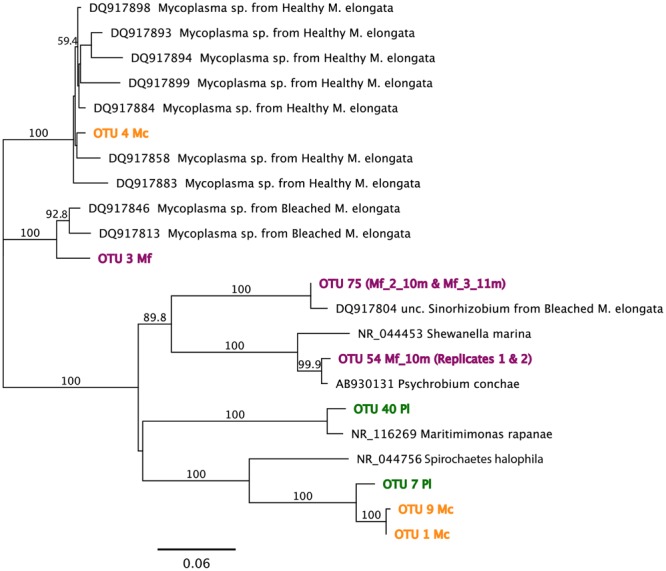
**Maximum-likelihood phylogenetic analyses for OTU representative sequences using the Jukes–Cantor model of substitution with 1000 bootstrap replicates (bootstrap values > 0.5 displayed).** Reference sequences obtained from both NCBI NR and Reference 16S ribosomal RNA databases. See text for specific sequence percent identities. Mf: *Muricea fruticosa*, Mc: *Muricea californica*, SW: sea water, Pl: *Parazoanthus lucificum*.

**FIGURE 5:**
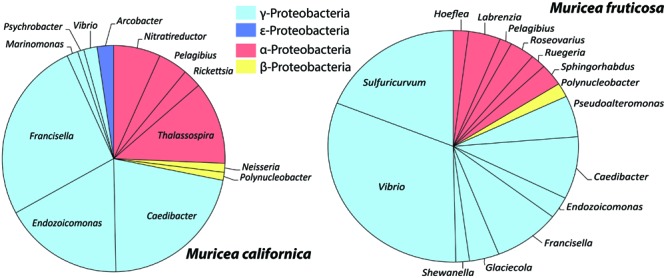
**Mean relative abundances of Proteobacteria taxa from *M. californica* and *M. fruticosa***.

Of the tested variables, host species type had the greatest effect in determining octocoral microbiome compositions accounting for 58.2% of the observed variability (PERMANOVA *F*_1,15_ = 19.5, *p* = 0.001). *Muricea* microbiomes all contained an average of 15% unclassified Bacteria compared to the SW and Pl samples where 2–10% of sequences were unclassifiable (**Figure 3**). Sequences clustered into OTU1 composed 6–64% of Mc microbiomes, compared to <0.001% of any other sampled microbiome, and were noticeably abundant in the Mc_12m and Mc_3_16m microbiomes. Phylogenetic analyses indicated OTU1 was at least 96% identical to OTU7 and 85% identical to NR_044756, *Spirochaeta halophila* (**Figure 4**). This observation is corroborated by our microscopic examinations of *Spirochaetes* in Mc mucus (**Figure 5**). OTU4 represented 6–61% of Mc microbiome sequences, vs. <0.3% in all other samples, while OTU3 composed 12–62% of Mf microbial communities, compared to <0.05% of all other sample microbiomes (**Figure 3**). OTUs 3 and 4 were 87.5% identical to each other, and representative sequences formed a monophyletic group with multiple *Mycoplasma* sequences isolated from *Muricea elongata*, a sister gorgonian species found in the coastal Gulf of Mexico and the Caribbean (NCBI PopSet: 134140623, Ranzer et al., 2007). OTU4, found in the Mc samples, formed a monophyletic group with *Mycoplasma* sequences isolated from healthy *M. elongata* colonies, while OTU3 grouped with those from bleached, diseased *M. elongata* colonies (**Figure 4**).

Overall, *Muricea* microbiomes had on average more γ-Proteobacteria sequences than α-Proteobacteria (*t* = 2.03, *df* = 80, *p* = 0.05; **Figure 5**), with the exception of Mf_3_11m, and Mf_2_10m, due to a large number of sequences from α-Proteobacteria OTU75. Other taxa showing *Muricea* spp. specificity included α-Proteobacteria such as *Thalassospira* (observed in 5 of 7 Mc samples, and no Mf samples), *Nitratireductor* (all Mc samples, no Mf sample), and γ-Proteobacteria like *Endozoicomonas, Caedibacter* and *Francisella* (observed in all *Muricea* samples, but higher abundances in Mc samples; *Caedibacter* and *Francisella* observed in 6 of 7 Mc samples; **Figure 5**). Also, *Vibrio* spp. (γ-Proteobacteria) sequences were observed in all *Muricea* samples, but more so in Mf samples (1–7% of Mf communities, <0.4% of Mc communities). *Candidatus nitrosopumilus* and *Sulfuricurvum* were observed in all Mf samples and no Mc samples (1–2% of mean Mf community composition). Overall, *Muricea* microbiomes had on average more γ-Proteobacteria sequences than α-Proteobacteria (*t* = 2.03, *df* = 80, *p* = 0.05; **Figure 5**), with the exception of Mf_3_11m, and Mf_2_10m, due to a large number of sequences from α-Proteobacteria OTU75. OTU75 was 98% identical to uncultured *Sinorhizobium*, a clonal sequence isolated from bleached *M. elongata* (**Figure 4**, NCBI PopSet: 134140623, Ranzer et al., 2007).

Variation between Mc colony microbiomes was significant (PERMANOVA: *F*_1,6_= 7.45, *R*^2^= 0.60, *p* = 0.025). Colony Mc_9m was distinct from other Mc colonies because more *Francisella* (γ-Proteobacteria) sequences were observed in each of the replicate branch communities (**Figure 3**, dendrogram). As previously stated, the high number of OTU1 sequences defined the Mc_12m microbiomes. Depth was not a significant factor in contributing to these observed differences.

Mf colony microbiomes were also significantly different from each other (PERMANOVA: *F*_1,6_= 4.33, *R*^2^= 0.38, *p* = 0.007). Colonies from 8 m and 11 m clustered together and separate from the Mf_10m colony. Approximately 80% of the 8-m and 11-m replicate branch microbiomes were dominated by OTU9 (6–17% of colony replicate branch microbiomes) and previously described OTU4 (**Figure 3**). OTU9 was 94% identical to OTU1, and >80% identical to NR_044576, *S. halophila*. OTU4 contributed to only 37% of Mf_10m microbiomes; OTU9 was minimally observed. This, and the relatively high abundance of Family NB1d sequences (γ-Proteobacteria OTU54, 99% identical to AB930131, *Psychrobium conchae*, **Figure 4**), and *Tenacibaculum* (Flavobacteria) sequences produced the clustering of branch replicate samples 1 and 2 from colony Mf_10m. Colony Mf_8m replicate branch microbiomes were distinct from Mf_11m because of the relatively high abundance of Sphingobacteria *Saprospiracea* (Bacteroidetes) and the Thaumarchaea *C. nitrosopumilus* (Marine Group I). Again, depth did contribute significantly in explaining this variation.

The sea water microbiome was different from all organism-associated microbiomes, dominated by α-Proteobacteria, namely *Candidatus* Pelagibacter (53% of microbiome) and γ-Proteobacteria SAR86 clade (12% of microbiome; **Figure 3**). These specific taxa minimally contributed (<0.13%) to all other microbiomes.

Pl microbiomes were also distinct. The relative abundance of OTU7 primarily distinguished the Pl microbiomes from all other samples (**Figure 3**; 21% of Pl_16m, 84% in Pl_9m, and less than 0.03% of any other sample communities), and most closely related to NR_044756, *Spirochaeta halophila* (85% identical, **Figure 4**). Pl_16m harbored a large number of Flavobacteriaceae sequences (**Figure 3**, 48% of community), specifically OTU40 (47.8% of community), which was 97% identical to NR_116269, *Maritimimonas rapanae* (**Figure 4**). OTU40 contributed <0.04% to any other microbiome, including Pl_9m.

**FIGURE 6:**
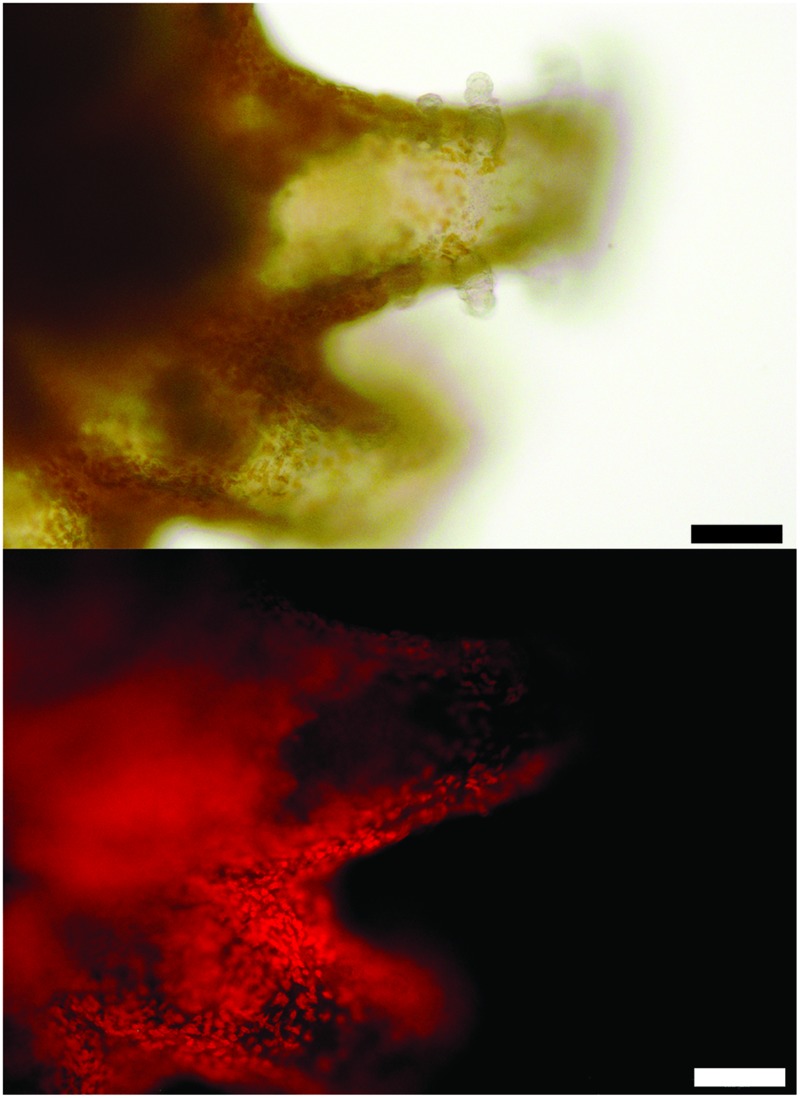
**A polyp of *M. californica* contains brown algae visualized with transmitted light (top) and fluorescence from blue light excitation (bottom, see Materials and Methods).** Scale bar: 100 μm.

Many features of the octocoral microbiomes were suggestive of photosymbiont-containing invertebrates including the observed strains of *Mycoplasma*, the presence of *Endozoicomonas*, and a higher γ-:*α-*Proteobacteria ratio in Mc samples. Thus, we examined the polyps of both species of *Muricea* using transmitted light and epifluorescence microscopy to determine if photosynthesizing algae were present. Indeed, 5-15 μm brown cells were observed in abundance within Mc polyps and fluoresced red when excited with blue light, indicating that the cells contain chlorophyll (**Figure 6**). Pigmented cells were not observed in Mf polyps (data not shown).

**FIGURE 7:**
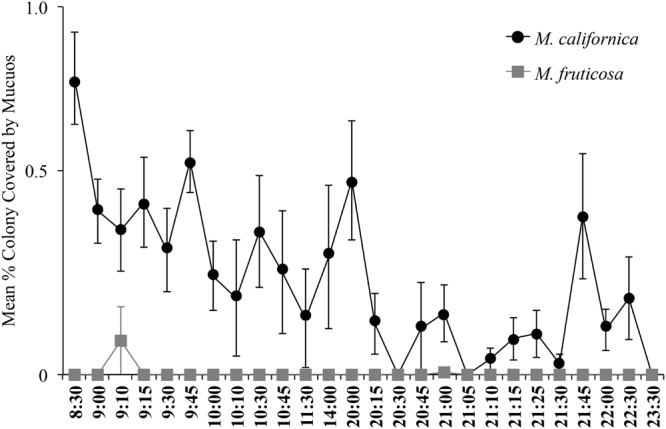
**Mucous coverage was almost continuously observed on *M. californica* (*n* = 4 colonies) and little to not at all on *Muricea fruticosa* (*n* = 2 colonies).** Colonies were observed daily from June 6, 2013 to September 19, 2013. Means were calculated from 2 to 24 total observations, depending on the time point.

Mc colonies produced mucus daily and, in general, mucus production appeared to be greater during daylight hours. Mucus was almost never observed on Mf colonies (**Figure 7**). Spirillum-shaped bacteria were observed in mucus from Mc (**Supplemental Figure S1**).

## Discussion

The microbiomes of two co-occurring and ecologically important temperate gorgonian octocoral species, *M. californica* and *M. fruticosa*, were compared to overlying sea water and nearby zoanthid associated microbiomes. We confirmed that host-associated microbial assemblages exist and are distinct from those in surrounding sea water (**Figure 3**) and other nearby benthic organisms (*P. lucificum*). Using inter- and intra-colony replication, we also observed that the microbiomes of each species of *Muricea* each have specific and predictable compositions.

Our results revealed specific relationships between hosts and members of their associated microbial communities. Both species of *Muricea* had an abundance of sequences from the bacterial phylum, *Tenericutes*, specifically from the genus, *Mycoplasma*. This relationship has been observed in deep-sea gorgonians (Gray et al., 2011), the cold water coral, *Lophelia pertusa* (Kellogg et al., 2009), and a species of gorgonian from the Atlantic Ocean, *Muricea elongata* (Ranzer et al., 2007). The data of Ranzer et al. (2007) also showed two strains of *Mycoplasma* associating separately with different colonies of *M. elongata*, but the distinction between those colonies was healthy and bleached. We saw a similar relationship: *Mycoplasma* strains associated with healthy *M. elongata* were also found in photosymbiont-containing *M. californica* microbiomes, and *Mycoplasma* strains associated with bleached *M. elongata* were observed in white polyp *M. fruticosa* microbiomes.

*Mycoplasma* requires secondary metabolite sterols and fatty acids (FAs) for growth (Ludwig et al., 2010). Such compounds are produced by *Muricea* (Popov et al., 1983; Gutiérrez et al., 2006), but the types of FA produced are highly dependent on the coral diet: FA produced by and transferred from symbiotic photosynthetic algae to the host coral are compositionally different than those produced by the coral’s own biosynthesis pathways (Imbs et al., 2007). This and our observation of chlorophyll-containing cells in the polyps of *M. californica* leads us to hypothesize that different strains of *Mycoplasma* consistently associate with either *M. californica* or *M. fruticosa* because of the different FA each produce.

Another major distinction between the microbial communities of co-occurring *Muricea* species was the greater abundance of sequences from the phylum *Spirochaetes* in *M. californica* samples. The genera *Spirochaeta* are chemoheterotrophic and can thrive in a variety of environments (Ludwig et al., 2010). *Spirochaetes* have been observed in other cnidarian groups including the cold-water coral *Lophelia* (Kellogg et al., 2009), hydra *Hydra attenuata* (Hufnagel and Myhal, 1977), and the mucous of sea pens *Pennatula phospherea* and *Pteroides spinosum* (Porporato et al., 2013). We observed *Spirochaetes*-like bacteria in the mucus of *M. californica* (**Supplemental Figure S1**) and hypothesize that the greater relative abundance of sequences in *M. californica* samples may be due to the daily mucus production and sloughing performed by this species, which was not been observed in *M. fruticosa*.

*Muricea* microbiomes consisted of relatively more *γ-Proteobacteria* sequences than *α-Proteobacteria.* Recently, Bourne et al. (2013) showed that photosymbiont-containing marine invertebrates had a greater abundance of *γ-Proteobacteria.* Those without photosymbionts generally maintained relatively more *α-Proteobacteria.* The photosymbionts observed in *M. californica* polyps may contribute to the abundance of *γ-Proteobacteria* sequences observed. The *γ-Proteobacteria* genera from *M. californica* samples were limited to *Endozoicomonas, Caedibacter*, and *Francisella*. *Endozoicomonas* sequences have been isolated from photosymbiont-containing corals and gorgonians (Bayer et al., 2013; Bourne et al., 2013) and metabolize the organic sulfur compound, dimethylsulfoniopropionate (DMSP), a byproduct of photosynthetic algae (Raina et al., 2009). The observed photosymbionts may be producing DMSP and therefore influencing the presence of *Endozoicomonas*. Interestingly, *M. fruticosa* had similar *γ-Proteobacteria* genera but in lower relative abundances. *Vibrio* sequences were the most abundant *γ-Proteobacteria* sequences found. Members of the genus *Vibrio* are among the known pathogens of corals (Bally and Garrabou, 2007; Mouchka et al., 2010; Vezzulli et al., 2010), and diseased corals tend to harbor greater microbial diversity compared to healthy conspecifics (Bourne et al., 2008; Sunagawa et al., 2009). While *M. fruticosa* colonies may simply harbor more microbial diversity naturally, the greater OTU diversity and number of *Vibrio* sequences, in addition to the particular strain of *Mycoplasma* observed could be indicative of microbial community instability and possibly a diseased state.

The sequences of nitrifying microbes found uniquely amongst the host-associated microbiomes of the *Muricea* were of particular interest. *M. fruticosa* samples contained the Archaeon *C. nitrosopumilus*, a known ammonia oxidizer (Francis et al., 2007), and *M. californica* had an abundance of *Nitratireductor*, a nitrate-reducing bacteria (Labbé et al., 2004). Nitrogen cycling in corals has been observed (Shashar et al., 1994), and a model of the role *Nitrosopumilus* may play in ammonia oxidation has been presented (Siboni et al., 2008). Nitrogenous waste (dissolved inorganic nitrogen, DIN) from coral colonies is either released into the surrounding sea water, in the case of azooxanthellate corals, or transferred to photosymbionts, if present (Dubinsky and Jokiel, 1994). In fact, the density, chlorophyll *a* content, and rate of photosynthesis of coral photosymbionts is tightly coupled to the availability of DIN (Hoegh-Guldberg and Smith, 1989). Thus, *Muricea* metabolic waste products may offer a distinct niche for the observed nitrifying microbes, and the presence of the chlorophyll-containing cells may impact this diversity by altering the amounts and types of DIN released.

Interestingly, the gorgonian samples both contained a high number of novel sequences, those that were unclassifiable beyond the domain level, because there were no reference sequences greater than 80% identical. This proportion of sequences implies that these corals may be important sources of novel biological diversity. Gorgonians have been targeted as promising sources of novel secondary metabolites or marine natural products (MNPs; Rocha et al., 2011). But in most cases, an associated microbe, and not the invertebrate host, is producing the compound(s) of interest (Rath et al., 2011) and our study offers novel information about the microbial organisms associated with gorgonians that may be relevant targets for bio-discovery.

Gorgonian microbiomes, in general, are extremely understudied, especially compared to their scleractinian relatives. While a 16S rRNA gene diversity study is not definitive of functional or metabolic interactions in a metaorganism, a comparative approach to microbial diversity, as we have described here, can highlight and substantiate significant differences between the available niches provided by various host organisms.

## Author Contributions

JH and KH designed the experiment. JH collected, prepared, and analyzed the samples. JH produced the figures and wrote the manuscript. KH advised on the figure production as well as the manuscript preparation.

## Conflict of Interest Statement

The authors declare that the research was conducted in the absence of any commercial or financial relationships that could be construed as a potential conflict of interest.

## Supplementary Material

FIGURE S1 **Muricea californica mucous contains Spirochaetes-like cells.** Mucus 30 from M. californica stained with SYBR Gold and examined using fluorescence microscopy.
