# Supplementary material for: Microbiomes of Muricea californica and M. fruticosa: Comparative Analyses of Two Co-occurring Eastern Pacific Octocorals
Source: Front Microbiol. 2016 Jun 21;7:917. doi: 10.3389/fmicb.2016.00917 (PMC4914490; doi:10.3389/fmicb.2016.00917)
Supplement: Supplementary file 1 [file Table_1.DOCX]

Supplemental Table 1. Sample sequence statistics.

|  | Before Sample Normalization | | After Sample Normalization | | |
| --- | --- | --- | --- | --- | --- |
|  | No. Sequences | No. OTUs (97%) | No. Sequences | No. OTUs (97%) | Good's Coverage |
| Mc_1_9m | 29168 | 535 | 10448 | 473 | 0.955 |
| Mc_2_9m | 25848 | 421 | 10448 | 345 | 0.967 |
| Mc_1_12m | 10448 | 224 | 10448 | 287 | 0.973 |
| Mc_2_12m | 19491 | 178 | 10448 | 173 | 0.983 |
| Mc_1_16m | 31340 | 394 | 10448 | 279 | 0.973 |
| Mc_2_16m | 29257 | 375 | 10448 | 301 | 0.971 |
| Mc_3_16m | 25601 | 411 | 10448 | 354 | 0.966 |
| Mf_1_8m | 27709 | 405 | 10448 | 353 | 0.966 |
| Mf_2_8m | 28196 | 427 | 10448 | 356 | 0.966 |
| Mf_3_8m | 24459 | 534 | 10448 | 552 | 0.947 |
| Mf_1_10m | 22877 | 330 | 10448 | 354 | 0.966 |
| Mf_2_10m | 15643 | 629 | 10448 | 804 | 0.923 |
| Mf_3_10m | 27921 | 453 | 10448 | 384 | 0.963 |
| Mf_1_11m | 19884 | 418 | 10448 | 408 | 0.961 |
| Mf_2_11m | 33225 | 584 | 10448 | 437 | 0.958 |
| Mf_3_11m | 12308 | 405 | 10448 | 537 | 0.949 |
| Pl_9m | 11291 | 169 | 10448 | 221 | 0.979 |
| Pl_16m | 23701 | 241 | 10448 | 283 | 0.973 |
| SW_12m | 17348 | 709 | 10448 | 1733 | 0.834 |
