# Supplementary figures and images for: Microbiomes of Muricea californica and M. fruticosa: Comparative Analyses of Two Co-occurring Eastern Pacific Octocorals
Source: Front Microbiol. 2016 Jun 21;7:917. doi: 10.3389/fmicb.2016.00917 (PMC4914490; doi:10.3389/fmicb.2016.00917)

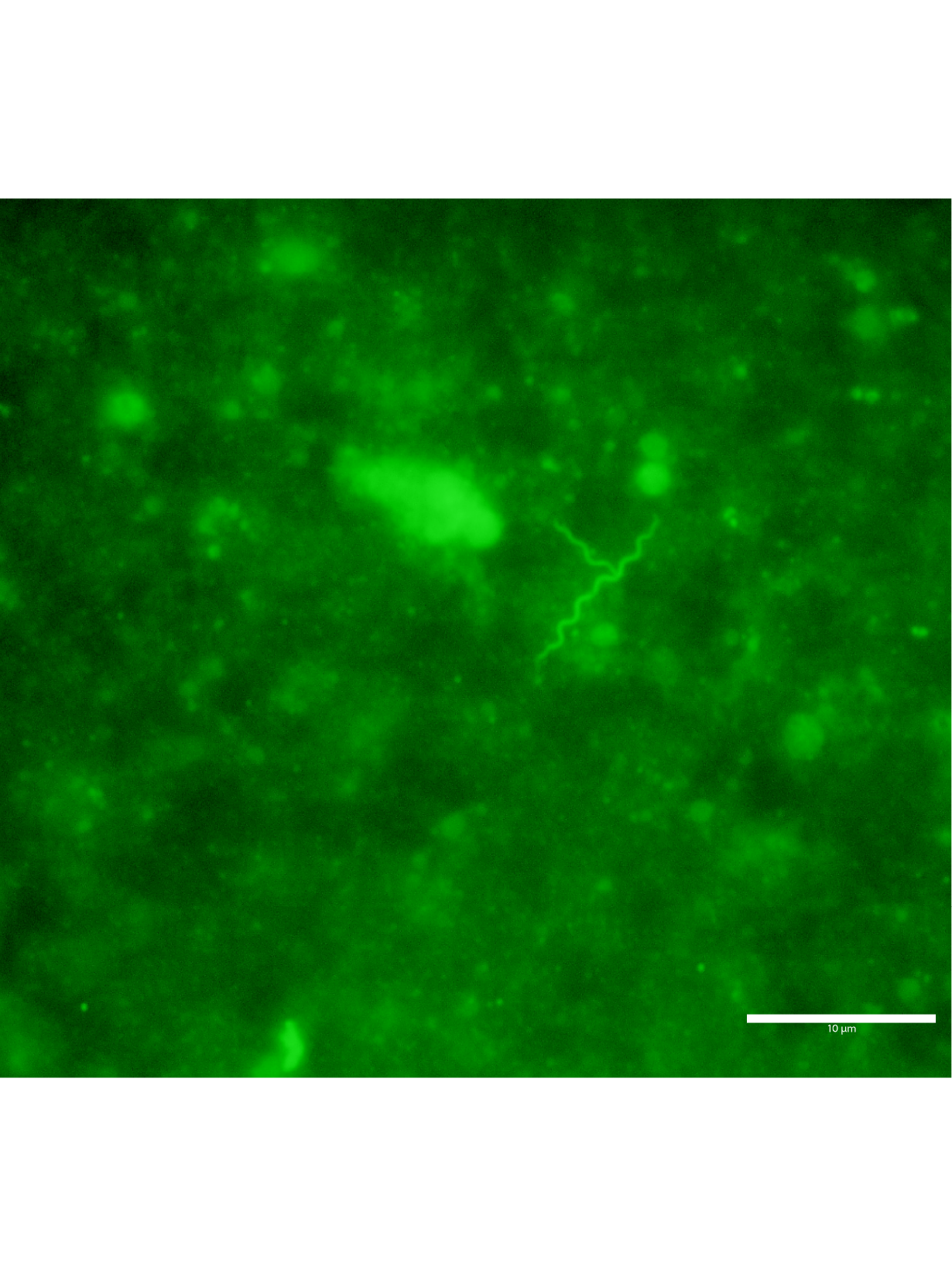

Supplement: FIGURE S1 — Muricea californica mucous contains Spirochaetes-like cells. Mucus 30 from M. californica stained with SYBR Gold and examined using fluorescence microscopy. [file Image_1.TIF]
